# Core Outcomes of Self-Care Behaviours in Patients with Breast Cancer Treated with Oral Anticancer Agents: A Systematic Review

**DOI:** 10.3390/cancers16234006

**Published:** 2024-11-29

**Authors:** Silvia Ucciero, Federica Lacarbonara, Angela Durante, Francesco Torino, Katarzyna Lomper, Ercole Vellone, Marco Di Nitto

**Affiliations:** 1 Department of Biomedicine and Prevention, Tor Vergata University of Rome, Via Montpellier 1, 00133 Rome, Italy; ercole.vellone@uniroma2.it; 2 Directorate of Health Professions, School of Nursing, University Hospital of Padua, 35128 Padua, Italy; federica.lacarbonara@aopd.veneto.it; 3 School of Advanced Studies Sant’Anna, Health Science Center, 56127 Pisa, Italy; 4 Fondazione Toscana “Gabriele Monasterio”, 56124 Pisa, Italy; 5 Department of Systems Medicine, Medical Oncology, Tor Vergata University of Rome, 00133 Rome, Italy; torino@med.uniroma2.it; 6 Department of Nursing, Faculty of Nursing and Midwifery, Wroclaw Medical University, 50-996 Wrocław, Poland; katarzyna.lomper@umw.edu.pl; 7 Department of Health Sciences, University of Genoa, Via Antonio Pastore 1, 16132 Genoa, Italy; marco.dinitto@unige.it

**Keywords:** self-care, oral anticancer agents, cancer, self-management, adherence, outcome, breast cancer

## Abstract

Background/Objectives: The use of oral anticancer agents (OAA) dates to the late 20th century in cancer treatment. It is crucial that patients implement self-care behaviours to keep their disease stable and manage their OAA treatment. The three dimensions of self-care according to Riegel et al., self-care maintenance, self-care monitoring, and self-care management, may be implemented to avoid negative outcomes. This paper seeks to identify outcomes associated with self-care in breast cancer patients during treatment with OAA and to compare which of these outcomes fall into the core outcome categorizations in oncology (minimal set of outcomes that research on a given health issue should measure). Methods: A systematic review with narrative synthesis was conducted. This study included patients with breast cancer taking any kind of OAA and described outcomes of self-care. The search was performed on MEDLINE, Web of Science and CINAHL/PsycINFO; Results: Of 4173 records, eight studies were selected and reviewed. The core outcomes mainly considered were mortality, survival, disease recurrence and quality of life. All studies focused only on pharmacological adherence outcome; none of them focused on other dimensions of self-care. Conclusions: This systematic review highlighted that there is a great lack of research on outcomes related to self-care in patients with breast cancer taking OOA. Even though pharmacological adherence to OAA is important, other behaviours are also important to improve patients’ outcomes, but they have not been studied. Further research is needed to study how self-care behaviours can impact patients’ outcomes.

## Simple Summary

The use of oral anticancer agents (OAAs) is increasing, and even more people need to monitor and manage their treatment pathway. A clear map of self-care outcomes studied so far can provide insights for clinical practice and future studies. The study revealed that all included articles considered, as intervention, treatment adherence and, as outcomes, mortality, survival, disease recurrence and quality of life. Adherence to OAA treatment has been found to reduce mortality and increase survival. However, important outcomes such as economic, social or psychological outcomes should be assessed in future studies to provide a complete picture of improvements that can be derived from self-care behaviours.

## 1. Introduction

Since the late 20th century, oral anticancer agents (OAA) have been increasingly used among standard treatments for patients affected by the most frequent cancer types [1,2]. The success of OAA is due not only to their efficacy and tolerability but also to their flexibility. OAAs indeed allow patients to maintain their daily routine, taking the anticancer treatment at home and avoiding frequent hospital visits, compared to intravenous treatments [3,4]. However, patients on OAAs can adhere to treatment poorly [5,6] and suffer from severe side effects [7]. Thus, it appears crucial that patients collaborate closely with their healthcare professionals to understand OAA risks, benefits, and proper administration, along with appropriate prevention and management of side effects. To achieve these aims, patients should adopt self-care behaviours to reach the best disease control and the optimal management of the OAA treatment [8].

Self-care is defined as the process by which the patient seeks to ensure physical, physiological and emotional stability in the presence of an illness (self-care maintenance), monitors the possible appearance of signs and symptoms related to the illness (self-care monitoring) and takes action if they recognise elements of relapse or worsening (self-care management) [9]. Self-care has been found to be associated with several positive outcomes related to patients with chronic diseases, particularly in patients with chronic diseases such as heart failure [10] and type 2 diabetes mellitus [11]. Self-care can improve patient quality of life [12], and can reduce mortality rate [13,14,15], hospitalisations [16], and unplanned access to care [17]. Similarly, in the case of patients with cancer, several studies have shown that inadequate self-care behaviours (e.g., non-adherence to treatment) can lead to poorer outcomes, such as worse symptom burden [18] and quality of life [19], disease recurrence, increased inpatient days, higher overall health care spending, and lower disease-free survival [20,21,22]. However, literature that identified outcomes of self-care behaviours in cancer is sparse and has never been appraised systematically. Specifically, in cancer care, several core outcomes have been identified that should be considered when assessing patients with cancer, but it is unknown how self-care can influence those.

The concept of core outcomes (COs) has emerged as a vital framework for assessing the effectiveness and impact of cancer care. Core outcomes have been defined as minimum sets of outcomes that, whether applied in different situations or in clinical studies for a certain illness condition, should be measured and reported [23]. COs in oncology encompass a set of key measures that serve as fundamental indicators of treatment success, patient well-being, and healthcare system performance. CO sets serve as a universal language, providing standardised tools for researchers, clinicians, and patients to assess the effectiveness and value of cancer treatments. Focusing on core outcomes allows better comparison of different interventions, tailoring treatments to individual patient needs, and improving the quality and delivery of cancer care [24,25]. Meregaglia et al. and Ramsey et al. [24,25] provided a detailed overview of COSs developed for use with oncology populations. The COs identified in the oncology field are listed in the Table 1.

## 2. Materials and Methods

### 2.1. Study Design

A systematic review with narrative synthesis was conducted [26]. The protocol was registered with the International Prospective Register of Systematic Reviews (PROSPERO) on 13 February 2022 (CRD42022299684, available from https://www.crd.york.ac.uk/prospero accessed on 13 July 2024). The reporting of this review was performed in accordance with the Preferred Reporting Items of Systematic Reviews and Meta-Analyses (PRISMA) guidelines [27].

### 2.2. Search Strategy

A preliminary search was performed in MEDLINE (PubMed) to find relevant keywords and adapt the search based on preliminary results. After refinement of the search string, a complete search was performed in the databases MEDLINE (PubMed), Web of Science (Clarivate), Cumulative Index of Nursing and Allied Health—CINAHL (EBSCOhost) and PsycINFO (EBSCOhost) from inception until the end of July 2023. Subsequently, an update of the search string was conducted in July 2024. Medical Subject Headings (MeSH) and free text search terms were employed in the search strategy across all databases.

Selected keywords were related to self-care (e.g., ‘self-care’, ‘self-management’ and ‘adherence’, ‘self-monitoring’), anticancer therapy (e.g., ‘antineoplastic agent’, ‘oncolytic agent’ and ‘targeted medicines’), predictors and outcomes (‘predictor’, ‘self-care determinants’, ‘outcomes’) and cancer (e.g., ‘neoplasm’, ‘tumour’ and ‘cancer’). Moreover, only studies that were focused on outcomes of self-care were considered in this article, as other predictors have been published elsewhere [28]. The complete search strategy is available as Supplementary Material (Table S1).

### 2.3. Eligibility Criteria

In this review, the following eligibility criteria were adopted: (a) studies conducted in adult patients (>18 years) with solid cancer in any anatomical site, excluding melanoma, receiving any kind of OAA; (b) studies reporting outcomes of self-care behaviours; (c) articles in English, Italian and Spanish; and (d) articles reporting primary quantitative studies. The following criteria for exclusion were applied: (a) articles that included patients treated with oral and intravenous anticancer medicines; (b) studies discussing the perspectives of medical experts; and (c) editorials, letters, and reviews.

### 2.4. Screening and Selection

All pertinent studies that were found in each database were first imported into Rayyan^®^ [29]. After removing duplicates, eligibility was independently verified by two researchers (FL and SU) using the title and abstract of each record. Any disagreement was resolved through discussion. In the absence of agreement, a third researcher (MDN) was consulted to reach an agreement.

### 2.5. Evaluation of Methodological Quality

Studies with all types of designs were assessed using the Joanna Briggs Institute [30] agreement tool. This instrument is meant to be used as a checklist to evaluate a study’s methodological quality and ascertain how well it handled the potential for bias in its planning, execution, and analysis [30].

Based on the assessment performed, a percentage score was assigned to each study and this allowed us to classify the papers based on their methodological evaluation. This score was computed by considering the proportion of the total number of items from the JBI checklist used.

Each article received a percentage score based on the methodological review, and was categorised as follows: studies of low quality (score = 0–45%), moderate quality (score = 54–75%), high quality (score = 82–91%), very high quality (score = 100%).

### 2.6. Data Extraction and Synthesis

One researcher (SU) extracted relevant data, and another (FL) checked the accuracy of these findings. The data extraction process was carried out using a spreadsheet. Any disagreement was settled through discussion.

The author and year of publication, study design, anatomical site of cancer, type of OAA, core outcome, geographic location, and sample size were the fields extracted from the included studies. Measures of effect size of the study’s findings were extracted, including odds ratios (OR), hazard ratios (HR), risk ratios (RR), regressions estimates, mean differences with confidence intervals (CI), and *p*-values. Results are presented in tabular format and using narrative synthesis to obtain a more comprehensive reading of extracted data.

## 3. Results

### 3.1. Study Selection

**Figure 1:**
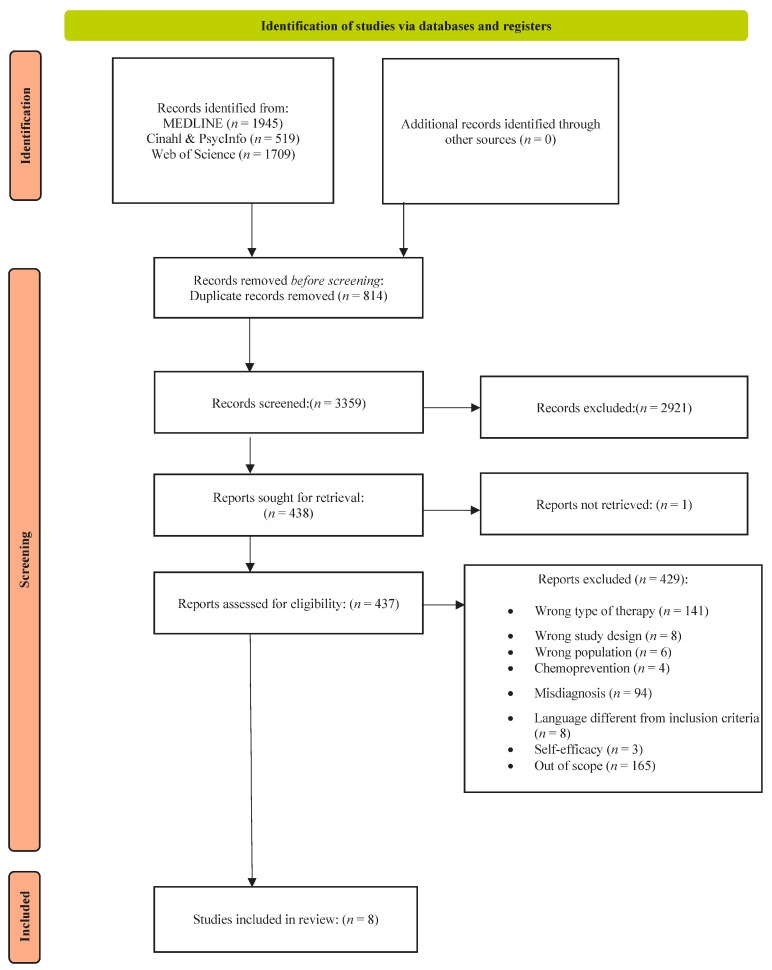
Flow chart—PRISMA.

Four thousand, one hundred and seventy-three records were retrieved from the database searched. After the duplicates were removed 3359 records were screened for relevance by title and abstract. Among the 437 full texts examined, eight studies were included and 429 were excluded [27] (Figure 1).

### 3.2. Study Characteristics

Eight studies that reported outcomes of patients with breast cancer using OAAs were included. A summary of the study characteristics is shown in Table 2. All studies [22,31,32,33,34,35,36,37] considered patients with breast cancer, even if the study protocol wanted to investigate core outcomes assessed for patients with any types of cancer. Moreover, all studies considered specifically outcomes of adherence to OAAs (considered one of the self-care maintenance behaviours). The OAAs considered in the included studies were tamoxifen, selective oestrogen receptor modulators (SERMs) or aromatase inhibitors (AIs). Three studies were conducted in the USA [32,34,36], two in the UK [22,31], one in Canada [33], one in Germany [35] and one in China [37].

### 3.3. Methodological Quality

The results of the JBI’s critical appraisal tools are resented in Tables S2–S4. One study obtained a low-quality score (score = 36%), two studies obtained high quality (score = 91%) and five obtained very high quality (score = 100%).

### 3.4. Outcome of Self-Care

Of the eight selected articles, all reported only self-care maintenance behaviours (e.g., outcomes related to OAA adherence). No eligible articles addressed self-care monitoring or self-care management dimensions. This study mapped self-care outcomes in breast cancer patients taking OAA and categorised them according to the taxonomy (Table 3) used by Meregaglia et al. and Ramsey et al. [24,25].

Five studies [32,33,34,35,37] investigated core outcomes that can be categorised under “mortality and survival”. Outcomes such as mortality and survival belong to this category. One study [34] analysed the outcome of mortality, reporting that patients without adequate adherence to OAA had significantly higher risk of death. Four studies analysed patient survival [32,33,35,37]. Chirgwin et al. concluded that patients who had low adherence to OAA had a lower likelihood of disease-free survival [32]. Davies et al. concluded that patients that were able to adhere to OAAs continuously for at least three years had better survival outcomes [33]. Xu reported that the men with breast cancer who adhered to tamoxifen had longer survival and disease-free survival at five and ten years after diagnosis [37], and Yuan et al. found that patients who were adherent to endocrine therapy had significantly worse overall survival but a non-significant change in breast-cancer-specific survival [35].

Three studies can be categorised as “outcomes related to neoplasms”, as they analysed the probability of disease recurrence [22,31,36]. Women with breast cancer who were not adherent to adjuvant endocrine therapy had a significantly higher risk of cancer recurrence than women who were adherent to therapy [31]. The study by Chang et al. showed that women under endocrine therapy in breast cancer who discontinued therapy before six months, compared with those with near-perfect adherence, were associated with a higher risk of breast cancer recurrence [36]. McCowan et al. reported that women with breast cancer treated with tamoxifen who had lower adherence to treatment experienced disease recurrence in a shorter time frame [22]. One study [22] can be categorised under the “global quality of life” CO category. The results of McCowan et al. (2013) [22] showed that poor adherence was significantly correlated with poorer quality of life. No studies assessed any other COs.

## 4. Discussion

This review, with its unique focus on self-care-related outcomes, aimed to identify and classify these outcomes in the population of breast cancer patients under treatment with OAA. It also sought to understand whether, in the literature, self-care-related outcomes have ever been correlated with COs in oncology. This systematic review is the first attempt to understand whether and how self-care-related outcomes were studied in patients under treatment with OAA, and it provides a detailed overview of these outcomes in this population.

The main result of this review was that the included studies focused only on treatment adherence (which can be considered only part of self-care behaviours) and the core outcomes mainly considered were mortality [34], survival [32,33,35,37], disease recurrence [22,31,36], and quality of life [22]. Specifically, treatment adherence is closely related to reduced risk of mortality and disease recurrence, increased survival, and improved quality of life. The focus on these specific core outcomes can also be explained by their importance, as these outcomes are often prioritised in clinical research due to their immediate relevance to patient health and healthcare policy [38]. The consequences of low adherence to OAA are poorer health outcomes, increased healthcare costs, and worse quality of life [22,31,32,33,34,35,36,37]. Of note, one study [35] reported a decreased likelihood of survival for patients adherent to therapy, but this result could be due to patient survival itself, as patients in the group with lower survival had less time to be adherent to medication.

Many COs have not been evaluated (Table 3). Surprisingly, none of the included studies evaluated symptom burden (i.e., pain, fatigue, bone pain, weight loss, anaemia, or performance status, categorised as “general outcomes”), in contrast to the most recent research trends in the field of oncology, which aim to make an in-depth study of symptoms for the identification of clusters of patients with similar behaviours and outcomes, to tailor appropriate interventions [39,40]. It is essential to study treatment outcomes to ensure high-quality healthcare. Particularly in the field of oncology, ensuring high quality care involves providing patient-centred care, which is considered a central aspect of cancer care. Therefore, it is crucial to study the factors that influence the success of treatment, patient well-being and the effectiveness and efficiency of healthcare system performance [41]. Moreover, the COs analysed so far may not have considered what are patient-sensitive outcomes. Indeed, outcomes such as survival and mortality are essential from a clinical point of view and clearly important both as proxies for an appropriate treatment outcome and patient outcomes. However, other COs, such as financial distress and cost concerns (economic outcome) or quality of life, are considered essential in cancer patients because costs, such as loss of income, can have a negative impact on both patients and their families. Evaluation of these COs is essential to assess the impact of self-care behaviours, and these outcomes can be measured through specific Patient-Reported Outcome Measures, as this discomfort can result in reduced adherence to treatment [42].

Another significant finding of this review was that all included studies focused on breast cancer patients, although the search strategy was intended for all cancer sites. This result is particularly relevant given that breast cancer is the second most common cancer worldwide, underscoring the global need for more studies on patients affected by various cancer types, especially considering that OAA are often among standard therapeutic options [43]. This gap should be filled with future studies. Moreover, regardless of tumour type, studies on OAA should also consider the disease burden, as self-care behaviours can vary based on disease burden, as in the management of OAAs, leading to differences in patient outcomes.

Furthermore, it is important to note that the COs identified were only studied in correlation with adherence to OAAs. Adherence can be considered just one aspect of the self-care process, specifically falling under the category of self-care maintenance behaviours required to maintain stable disease [44]. However, patients taking OAA also need to monitor symptoms related to cancer and treatment side effects (self-care monitoring) and to manage these symptoms and side effects (self-care management). Unfortunately, research on the effects of self-care monitoring and self-care management on COs is currently lacking. Furthermore, the unique focus on adherence has overlooked the crucial role of informal caregivers in OAA treatment, which is essential for supporting patients’ self-care behaviours [45]. This underscores the need for further research in these areas. Indeed, previous literature found that caregiver contributions to the self-care of patients with chronic diseases can impact patient outcomes. Patients performing better self-care have a better quality of life [12,46], reduced mortality [13,14,15], reduced hospitalisations [16], and emergency room admissions [17]. Moreover, the caregiver role has also been outlined for patients on OAAs [45]. Thus, self-care in patients taking OAAs has not yet been studied in its full complexity. This lack of knowledge could hide possible benefits for patients on OAAs that perform adequate self-care behaviours, and more research in this field is needed.

The results of this review suggest the need for a comprehensive assessment of COs in studies in the oncology field. The included articles used different selection criteria and processes for assessing outcomes, which were often not well described nor justified. Moreover, all COs in this review were analysed from data cohorts without considering patient perception.

### 4.1. Implications for Future Research

This review outlined several implications for future research. First, further studies should aim to incorporate a broader range of outcomes to fully capture the impact of self-care behaviours, specifically studying all those COs related to patient functioning (e.g., physical, cognitive, etc.) that can be improved by adequate self-care behaviours. Moreover, standardising the measurement and reporting of diverse outcomes across studies can enhance comparability and synthesis of evidence. This could involve developing consensus-based core outcome sets for self-care behaviour research.

Another aspect that needs attention in future research is evaluation of the economic outcomes of self-care interventions, which still need to be evaluated. Adequate self-care behaviours can lead to less use of inappropriate healthcare services (such as emergency room or rehospitalisation), with important economic implications that have not been evaluated so far. Indeed, healthcare systems are increasingly focused on the homecare setting, and appropriate self-care behaviours can promote continuous care at home, limiting, for example, inappropriate emergency room use.

This aspect is also of considerable importance in terms of economic implications, as inappropriate use of healthcare services can negatively impact the healthcare system’s economy, risking increasing inequalities in access to healthcare services for other patients with real care needs.

Finally, it is crucial to emphasise the need for further research that focuses on the various COs in the oncology field. This research can guide future research toward the development of a comprehensive framework for COs. A framework that encompasses a wide variety of outcomes can help ensure that all relevant aspects of self-care behaviours are systematically evaluated, providing a more in-depth understanding of patient health and wellbeing.

### 4.2. Limitations

This systematic review has limitations. First, despite efforts to exhaustively search for relevant studies, it is possible that some studies may have been missed. Another limitation concerns the concept of self-care, which is still poorly explored in the literature. Only the concept of adherence, considered a component of self-care maintenance, was investigated.

## 5. Conclusions

This review showed a significant lack of research on COs related to cancer patients taking oral anticancer agents. Increased adherence to OAAs means reduced mortality and increased survival. Further longitudinal studies are needed to evaluate the COs of self-care in cancer patients on OAAs.

## Figures and Tables

**Table 1:** Core outcomes identified in the oncology field [24,25].

Mortality and survival (e.g., survival)
Outcomes related to neoplasms (e.g., disease progression)
Renal and urinary outcomes (e.g., urinary incontinence)
Metabolism and nutrition (e.g., nutritional status)
Musculoskeletal and connective tissue (e.g., arm symptoms)
Nervous system (e.g., neuropathy)
Gastrointestinal (e.g., diarrhoea)
Endocrine outcomes (e.g., hormonal symptoms)
Subcutaneous tissue and skin
Reproductive system outcomes (e.g., erectile/sexual dysfunction)
General outcomes (e.g., pain, fatigue)
Physical functioning
Cognitive functioning
Emotional functioning/wellbeing
Social functioning
Role functioning
Global quality of life
Economic outcomes (e.g., cost-effectiveness)
Need for intervention (e.g., need for salvage therapy)
Delivery of care (e.g., time to treatment failure)
Adverse events/effects (e.g., thromboembolic disease, major systemic therapy effects)

Currently, no study has explored which COs were associated with self-care in patients on OAA treatment. Therefore, the aim of this review was to synthetise the evidence regarding studies that identified the association between self-care behaviours in patients with breast cancer treated with OAA and COs in cancer care.

**Table 2:** Study characteristics.

Authors, Year, Country	Design	Sample Population	Name of the Anticancer	Main Themes and Findings Related to Outcome	JBI’s Critical Appraisal
Barron et al., 2013 UK [31]	Case control	1376 women with breast cancer (mean age 65 years)	SERMs or AIs	Women who were non-persistent with OAA had a significantly increased risk of recurrence of cancer (OR = 2.88; 95% CI = 1.11–7.46) in comparison to women who persisted with OAA.	100%
Chang et al., 2024 USA [36]	Retrospective	28,042 women with breast cancer (mean age 72 years)	TAM or AIs	Women who discontinued therapy before 6 months compared with those with nearly perfect adherence (HR = 1.84, 95% CI = 1.46–2.33) were associated with an increased risk of recurrence of breast cancer.	100%
Chirgwin et al., 2016 USA [32]	RTC	6193 women with breast cancer	TAM or Letrozole	Patients who had low adherence (early cessation of letrozole and a compliance score of <90%) to OAA had a lower likelihood of disease-free survival (HR: 1.45, 95% CI, 1.09–1.93; HR: 1.61, 95% CI, 1.08–2.38, respectively).	36%
Davies et al., 2022 Canada [33]	Retrospective	284 women with breast cancer (mean age 64.6 years)	TAM	Patients that were able to follow OAA on an uninterrupted basis for at least 3 years had better survival outcomes than patients who received OAA for more brief time intervals.	100%
McCowan et al., 2013 UK [22]	Cohort	1263 women with breast cancer (52.1% with age ≥ 60 years)	TAM	Higher adherence rate was estimated to reduce disease recurrence by 8.95% (95% CI = 11.01–6.89%) and deaths from breast cancer by 8.65% (95% CI = 10.69–6.57%); Higher adherence rate was associated with expected further life years (QALY) of 14.78 and expected quality-adjusted life years (discounted at 3.5%) of 11.43 compared with low adherence, which had expected quality-adjusted life years of 13.35 and 10.31, respectively; Patients with low adherence had a shorter time to recurrence, increased medical costs and worse quality of life.	91%
Winn et al., 2016 USA [34]	Cohort	9492 women with breast cancer (mean age 75 years)	TAM or AIs	Mortality was higher among patients with worse adherence (those who discontinued OAA earliest had a 40% increased risk of death; those who had a consistent decline in adherence over the year had a 25% increased risk of death).	100%
Yuan et al., 2020 German [35]	Cohort	552 elderly women with ER-positive breast cancer (mean age 80 years)	TAM or AIs	Elderly women with localised ER-positive breast cancer who were adherent to endocrine therapy had significantly worse overall survival (HR, 1.40; 95% CI 1.17–1.69; *p* < 0.001) but a non-significant change in breast-cancer-specific survival (HR 0.78, 95% CI 0.45–1.37, *p* = 0.392); The other two factors associated with worse survival were larger tumour size and more comorbidities; The type of endocrine therapy (tamoxifen vs. AIs) made no difference in the survival (HR 0.94, 95% CL 0.71–1.25, *p* = 0.672).	91%
Xu et al., 2012 China [37]	Cohort	116 men with breast cancer (mean age 62.8 years)	TAM	Patients adhering to tamoxifen, compared with patients with lower adherence, had longer survival (*p* = 0.008) and disease-free survival (*p* = 0.007) at five and ten years from diagnosis.	100%

Notes: AIs, aromatase inhibitors; CI, confidence interval; HR, hazard ratio; OAA, oral anticancer agent; SERMs, selective oestrogen receptor modulators; TAM, Tamoxifen.

**Table 3:** Classification of patient-reported outcomes included in patients under OAA treatment.

Core Outcomes in the Oncology Field [24,25]	Barron 2013 [31]	Chang 2024 [36]	Chirgwin 2016 [33]	Davies 2022 [31]	McCowan 2013 [22]	Winn 2016 [34]	Yuan 2020 [35]	Xu 2012 [37]
Mortality and survival			✓	✓		✓	✓	✓
Outcomes related to neoplasms	✓	✓			✓			
Global quality of life					✓			

Notes: outcomes investigated by the included studies are reported with a tick mark.

## Supplementary Materials

The following supporting information can be downloaded at: https://www.mdpi.com/article/10.3390/cancers16234006/s1, Table S1: Search strategy; Table S2: Methodological quality assessment of studies (cross-sectional and observational); Table S3: Methodological quality assessment of studies; Table S4: Quality assessment with new version of JBI check list for RCT studies.
