# Supplementary material for: Core Outcomes of Self-Care Behaviours in Patients with Breast Cancer Treated with Oral Anticancer Agents: A Systematic Review
Source: Cancers (Basel). 2024 Nov 29;16(23):4006. doi: 10.3390/cancers16234006 (PMC11640320; doi:10.3390/cancers16234006)
Supplement: Supplementary file 1 [file cancers-16-04006-s001.zip › cancers-3304467-supplementary.pdf]

Table S1. Search Strategy

Search summary from 2012 to 2024

**Database Name**	**Platform**	**Date Coverage**	**Date of Search**	**# of results**
1. MEDLINE	PubMed	From 01-01-2014	13-07-2024	1,741
2. Cinahl & PsycInfo	EBSCOHost	From 01-01-2014	13-07-2024	484
3. Web of Science core collection	Clarivate	From 01-01-2014	13-07-2024	1,532

Search summary from 2023 to 2024

**Database Name**	**Platform**	**Date Coverage**	**Date of Search**	**# of results**
1. MEDLINE	PubMed	From 01-01-2014	13-07-2024	204
2. Cinahl & PsycInfo	EBSCOHost	From 01-01-2014	13-07-2024	35
3. Web of Science core collection	Clarivate	From 01-01-2014	13-07-2024	177

Total Records (2012-2024) = 4,173
Total Records after deduplication = 3,359
Total Records after title/abstract screening = 437
Total records after full text reading =8

1. Pubmed

Date of Search:

#	Search string	# of results
1	("self-care"[MeSH Terms] OR "self management"[MeSH Terms] OR "self-care"[Title/Abstract] OR "self management"[Title/Abstract] OR "adherence"[Title/Abstract] OR "medication adherence"[MeSH Terms] OR "self-monitoring"[Title/Abstract])	278,518
2	((("clinical trial"[Publication Type] OR "clinical trials as topic"[MeSH Terms] OR "clinical trial"[All Fields] OR ("prospective studies"[MeSH Terms] OR prospective stud* OR "retrospective studies"[MeSH Terms] OR retrospective stud* OR "longitudinal studies"[MeSH Terms] OR longitudinal stud* OR "observational study"[Publication Type] OR "observational studies as topic"[MeSH Terms] OR "observational study"[All Fields] OR observational research OR experimental study))) OR (non randomized controlled trial)) OR ((("cross sectional studies"[MeSH Terms] OR cross sectional study) OR ("case control studies"[MeSH Terms] OR case control study)) OR ("cohort studies"[MeSH Terms] OR cohort study))	6,424,115
3	("Antineoplastic Agents"[MeSH Terms] OR "Antineoplastic Agents"[Title/Abstract] OR "anticancer medicines"[Title/Abstract] OR "anticancer medicine"[Title/Abstract] OR "chemotherapy"[Title/Abstract] OR "chemotherapies"[Title/Abstract] OR "antineoplastic drugs"[Title/Abstract] OR "antineoplastic drug"[Title/Abstract] OR "antineoplastic agents, hormonal"[MeSH Terms] OR "hormonal therapy"[Title/Abstract] OR "hormonal drugs"[Title/Abstract] OR "hormonal drug"[Title/Abstract] OR "oncolytic agent"[Title/Abstract] OR "oncolytic agents"[Title/Abstract] OR "anticancer immunotherapies"[Title/Abstract] OR "antineoplastic immunotherapy"[Title/Abstract] OR "anticancer immunotherapy"[Title/Abstract] OR "targeted drugs"[Title/Abstract] OR "targeted drug"[Title/Abstract])	889,741
4	("predictor*"[Title/Abstract] OR "predictors"[Title/Abstract] OR "risk factor*"[Title/Abstract] OR "self-care determinants"[Title/Abstract] OR "cause*"[Title/Abstract] OR "benefit*"[Title/Abstract] OR "effect*"[Title/Abstract] OR "impact"[Title/Abstract] OR "outcome"[Title/Abstract] OR "outcomes"[Title/Abstract] OR "adverse events"[Title/Abstract] OR "adverse event"[Title/Abstract] OR "Patient Outcome Assessment"[MeSH Terms] OR "Patient Outcome Assessment"[All Fields] OR "patient centered outcome research"[All Fields])	13,878,631
5	1 AND 2 AND 3 AND 4	2,420
6	1 AND 2 AND 3 AND 4 and date limit from 2012	1,741
7		
8		
9		
10		

2. Cinahl & PsychInfo

Date of Search:

#	Search string	# of results
1	MH ("Self Care+" OR "Self-Management") OR TI ("self-care" OR selfcare OR "self-management" OR adherence OR “self-monitoring”) OR AB ("self-care" OR selfcare OR "self-management" OR adherence OR “self-monitoring”)	205,769
2	((rct or clinical trial* or randomized controlled trial* or controlled clinical trial*) OR non randomized clinical trial*) OR ((longitudinal stud* OR retrospective stud* OR prospective stud* OR observational stud* or observational research) OR (cross sectional stud* or cross-sectional stud*) OR (case control stud* or case-control stud*) OR (cohort stud* or case control stud* or experimental stud*))	2,056,360
3	TI (predictor* or "risk factor" or cause* or predisposition or determinant* OR "self-care determinants" or cause) OR TI (outcome* or benefit* or effect* or impact or “adverse event” or “adverse events” OR "patient outcome assessment" OR "patient centered outcome research") OR AB (predictor* or "risk factor" or cause* or predisposition or determinant* OR "self-care determinants") OR AB (outcome* or benefit* or effect* or impact or “adverse event” or “adverse events” OR "patient outcome assessment" OR "patient centered outcome research")	5,240,832
4	TI ("Antineoplastic Agents" OR "anticancer medicines" OR "anticancer medicine" OR chemotherapy OR chemotherapies OR "antineoplastic drugs" OR "antineoplastic drug" OR "hormonal therapy" OR "hormonal drugs" OR "hormonal drug" OR "oncolytic agent" OR "oncolytic agents" OR "anticancer immunotherapies" OR "antineoplastic immunotherapy" OR "anticancer immunotherapy" OR "targeted drugs" OR "targeted drug") OR AB ("Antineoplastic Agents" OR "anticancer medicines" OR "anticancer medicine" OR chemotherapy OR chemotherapies OR "antineoplastic drugs" OR "antineoplastic drug" OR "hormonal therapy" OR "hormonal drugs" OR "hormonal drug" OR "oncolytic agent" OR "oncolytic agents" OR "anticancer immunotherapies" OR "antineoplastic immunotherapy" OR "anticancer immunotherapy" OR "targeted drugs" OR "targeted drug")	96,286
5	1 AND 2 AND 3 AND 4	694
6	1 AND 2 AND 3 AND 4 and date limit from 2012	484
7		
8		
9		
10		

2. Web of Science

Date of Search:

#	**Search string**	**# of results**
1	TS=("self-care" OR selfcare OR "self-management" OR adherence OR “self-monitoring”)	309,895
2	TS=(((rct or clinical trial* or randomized controlled trial* or controlled clinical trial*) OR non randomized clinical trial*) OR ((longitudinal stud* OR retrospective stud* OR prospective stud* OR observational stud* or observational research) OR (cross sectional stud* or cross-sectional stud*) OR (case control stud* or case-control stud*) OR (cohort stud* or case control stud* or experimental stud*)))	5,954,134
3	TS=(predictor* or "risk factor" or cause* or predisposition or determinant* OR "self-care determinants" or cause OR outcome* or benefit* or effect* or impact or “adverse event” or “adverse events” OR "patient outcome assessment" OR "patient centered outcome research")	24,890,491
4	TS=("Antineoplastic Agents" OR "anticancer medicines" OR "anticancer medicine" OR chemotherapy OR chemotherapies OR "antineoplastic drugs" OR "antineoplastic drug" OR "hormonal therapy" OR "hormonal drugs" OR "hormonal drug" OR "oncolytic agent" OR "oncolytic agents" OR "anticancer immunotherapies" OR "antineoplastic immunotherapy" OR "anticancer immunotherapy" OR "targeted drugs" OR "targeted drug")	632,722
5	1 AND 2 AND 3 AND 4	1,910
6	1 AND 2 AND 3 AND 4 and date limit from 2012	1,532

Table S2 - Methodological quality assessment of studies (cross-sectional and observational)

**Author & Year**	**Were the criteria for inclusion in the sample clearly defined?**	**Were the study subjects and the setting described in detail?**	**Was the exposure measured in a valid and reliable way?**	**Were objective, standard criteria used for measurement of the condition?**	**Were confounding factors identified?**	**Were strategies to deal with confounding factors stated?**	**Were the outcomes measured in a valid and reliable way?**	**Was appropriate statistical analysis used?**	**%**
Barron et al., 2013	1	1	1	1	1	1	1	1	100%
Chang et al., 2024	1	1	1	1	1	1	1	1	100%
Davies et al., 2022	1	1	1	1	1	1	1	1	100%

Table S3 - Methodological quality assessment of studies (cohort)

**Author & Year**	**Were the two groups similar and recruited from the same population?**	**Were the exposures measured similarly to assign people to both exposed and unexposed groups?**	**Was the exposure measured in a valid and reliable way?**	**Were confounding factors identified?**	**Were strategies to deal with confounding factors stated?**	**Were the groups/participants free of the outcome at the start of the study (or at the moment of exposure)?**	**Were the outcomes measured in a valid and reliable way?**	**Was the follow up time reported and sufficient to be long enough for outcomes to occur?**	**Was follow up complete, and if not, were the reasons to loss to follow up describ**	**Were strategies to address incomplete follow up utilized?**	**Was appropriate statistical analysis used?**	**%**

									**ed and explore d?**			
McCowan et al., 2022	1	1	1	1	1	1	1	1	1	1	0	91%
Winn et al., 2016	1	1	1	1	1	1	1	1	1	1	1	100%
Yuan et al., 2020	1	0	1	1	1	1	1	1	1	1	1	91%
Xu et al., 2012	1	1	1	1	1	1	1	1	1	1	1	100%

Table S4: Quality assessment with new version of JBI check list for RCT studies

Study Author: Chirgwin et al.	Study Title: Treatment Adherence and Its Impact on Disease-Free Survival in the Br International Group 1-98 Trial of Tamoxifen and Letrozole, Alone and in Sequence	Study Year: 2016

Internal Validity		Choice - Comments/Justification	Yes	No	Unclear	N/A
Bias related to selection and allocation						
+	Was true randomization used for assignment of participants to treatment groups?		☐	☐	☒	☐
2	Was allocation to treatment groups concealed?		☐	☐	☒	☐
3	Were treatment groups similar at the baseline?		☒	☐	☐	☐
Bias related to administration of intervention/exposure						
4	Were participants blind to treatment assignment?		☐	☐	☒	☐
5	Were those delivering the treatment blind to treatment assignment?		☐	☐	☒	☐
6	Were treatment groups treated identically other than the intervention of interest?		☒	☐	☐	☐
Bias related to assessment, detection and measurement of the outcome						
7	Were outcome assessors blind to treatment assignment?		Yes	No	Unclear	N/A
	Outcome 1		☐	☒	☐	☐
	Outcome 2-7		☐	☐	☐	☒

			Yes	No	Unclear	N/A
8	**Were outcomes measured in the same way for treatment groups?**		**Yes**	**No**	**Unclear**	**N/A**
	**Outcome 1**		☒	☐	☐	☐
	**Outcome 2-7**		☐	☐	☐	☒

9	**Were outcomes measured in a reliable way**		**Yes**	**No**	**Unclear**	**N/A**
	**Outcome 1**		☒	☐	☐	☐
	**Outcome 2-7**		☐	☐	☐	☒

Bias related to participant retention

10	**Was follow-up complete and if not, were differences between groups in terms of their follow-up adequately described and analyzed?**					
	**Outcome 1**		**Yes**	**No**	**Unclear**	**N/A**
	Result 1		☒	☐	☐	☐
	Result 2		☐	☐	☐	☒
	Result 3		☐	☐	☐	☒
	**Outcome 2-7**		**Yes**	**No**	**Unclear**	**N/A**
	Result 1		☐	☐	☐	☒

Statistical Conclusion Validity

11	Were participants analyzed in the groups to which they were randomized?					
	**Outcome 1**		**Yes**	**No**	**Unclear**	**N/A**
	Result 1		☒	☐	☐	☐
	Result 2		☐	☐	☐	☒
	Result 3		☐	☐	☐	☒
	**Outcome 2-7**		**Yes**	**No**	**Unclear**	**N/A**
	Result 1		☐	☐	☐	☒

12	Was appropriate statistical analysis used?					
	**Outcome 1**		**Yes**	**No**	**Unclear**	**N/A**
	Result 1		☒	☐	☐	☐
	Result 2		☒	☐	☐	☐
	Result 3		☒	☐	☐	☐
	**Outcome 2-7**		**Yes**	**No**	**Unclear**	**N/A**
	Result 1		☐	☐	☐	☒

			Yes	No	Unclear	N/A
13	**Was the trial design appropriate and any deviations from the standard RCT design (individual randomization, parallel groups) accounted for in the conduct and analysis of the trial?**		☐	☒	☐	☐

Overall appraisal: **Include: ☒** **Exclude: ☐** **Seek Further Info: ☐**

Comments:

PRISMA 2020 Checklist

Section and Topic	Item #	Checklist item	Location where item is reported
**TITLE**			
Title	1	Identify the report as a systematic review.	1
**ABSTRACT**			
Abstract	2	See the PRISMA 2020 for Abstracts checklist.	2
**INTRODUCTION**			
Rationale	3	Describe the rationale for the review in the context of existing knowledge.	3-5
Objectives	4	Provide an explicit statement of the objective(s) or question(s) the review addresses.	5
**METHODS**			
Eligibility criteria	5	Specify the inclusion and exclusion criteria for the review and how studies were grouped for the syntheses.	6
Information sources	6	Specify all databases, registers, websites, organisations, reference lists and other sources searched or consulted to identify studies. Specify the date when each source was last searched or consulted.	7
Search strategy	7	Present the full search strategies for all databases, registers and websites, including any filters and limits used.	6-7
Selection process	8	Specify the methods used to decide whether a study met the inclusion criteria of the review, including how many reviewers screened each record and each report retrieved, whether they worked independently, and if applicable, details of automation tools used in the process.	6-7
Data collection process	9	Specify the methods used to collect data from reports, including how many reviewers collected data from each report, whether they worked independently, any processes for obtaining or confirming data from study investigators, and if applicable, details of automation tools used in the process.	7
Data items	10a	List and define all outcomes for which data were sought. Specify whether all results that were compatible with each outcome domain in each study were sought (e.g. for all measures, time points, analyses), and if not, the methods used to decide which results to collect.	5
	10b	List and define all other variables for which data were sought (e.g. participant and intervention characteristics, funding sources). Describe any assumptions made about any missing or unclear information.	5
Study risk of bias assessment	11	Specify the methods used to assess risk of bias in the included studies, including details of the tool(s) used, how many reviewers assessed each study and whether they worked independently, and if applicable, details of automation tools used in the process.	n.a
Effect measures	12	Specify for each outcome the effect measure(s) (e.g. risk ratio, mean difference) used in the synthesis or presentation of results.	n.a
Synthesis methods	13a	Describe the processes used to decide which studies were eligible for each synthesis (e.g. tabulating the study intervention characteristics and comparing against the planned groups for each synthesis (item #5)).	6-7

Section and Topic	Item #	Checklist item	Location where item is reported
	13b	Describe any methods required to prepare the data for presentation or synthesis, such as handling of missing summary statistics, or data conversions.	6-7
	13c	Describe any methods used to tabulate or visually display results of individual studies and syntheses.	6-7
	13d	Describe any methods used to synthesize results and provide a rationale for the choice(s). If meta-analysis was performed, describe the model(s), method(s) to identify the presence and extent of statistical heterogeneity, and software package(s) used.	6-7
	13e	Describe any methods used to explore possible causes of heterogeneity among study results (e.g. subgroup analysis, meta-regression).	n.a
	13f	Describe any sensitivity analyses conducted to assess robustness of the synthesized results.	n.a
Reporting bias assessment	14	Describe any methods used to assess risk of bias due to missing results in a synthesis (arising from reporting biases).	n.a
Certainty assessment	15	Describe any methods used to assess certainty (or confidence) in the body of evidence for an outcome.	n.a
**RESULTS**			
Study selection	16a	Describe the results of the search and selection process, from the number of records identified in the search to the number of studies included in the review, ideally using a flow diagram.	8
	16b	Cite studies that might appear to meet the inclusion criteria, but which were excluded, and explain why they were excluded.	8
Study characteristics	17	Cite each included study and present its characteristics.	8
Risk of bias in studies	18	Present assessments of risk of bias for each included study.	n.a
Results of individual studies	19	For all outcomes, present, for each study: (a) summary statistics for each group (where appropriate) and (b) an effect estimate and its precision (e.g. confidence/credible interval), ideally using structured tables or plots.	12-13
Results of syntheses	20a	For each synthesis, briefly summarise the characteristics and risk of bias among contributing studies.	12-13
	20b	Present results of all statistical syntheses conducted. If meta-analysis was done, present for each the summary estimate and its precision (e.g. confidence/credible interval) and measures of statistical heterogeneity. If comparing groups, describe the direction of the effect.	n.a
	20c	Present results of all investigations of possible causes of heterogeneity among study results.	n.a

PRISMA 2020 Checklist

Section and Topic	Item #	Checklist item	Location where item is reported
	20d	Present results of all sensitivity analyses conducted to assess the robustness of the synthesized results.	n.a
Reporting biases	21	Present assessments of risk of bias due to missing results (arising from reporting biases) for each synthesis assessed.	n.a
Certainty of evidence	22	Present assessments of certainty (or confidence) in the body of evidence for each outcome assessed.	n.a
**DISCUSSION**			
Discussion	23a	Provide a general interpretation of the results in the context of other evidence.	16-18
	23b	Discuss any limitations of the evidence included in the review.	18
	23c	Discuss any limitations of the review processes used.	18
	23d	Discuss implications of the results for practice, policy, and future research.	18
**OTHER INFORMATION**			
Registration and protocol	24a	Provide registration information for the review, including register name and registration number, or state that the review was not registered.	5
	24b	Indicate where the review protocol can be accessed, or state that a protocol was not prepared.	5
	24c	Describe and explain any amendments to information provided at registration or in the protocol.	5
Support	25	Describe sources of financial or non-financial support for the review, and the role of the funders or sponsors in the review.	5
Competing interests	26	Declare any competing interests of review authors.	19
Availability of data, code and other materials	27	Report which of the following are publicly available and where they can be found: template data collection forms; data extracted from included studies; data used for all analyses; analytic code; any other materials used in the review.	

From: Page MJ, McKenzie JE, Bossuyt PM, Boutron I, Hoffmann TC, Mulrow CD, et al. The PRISMA 2020 statement: an updated guideline for reporting systematic reviews. BMJ 2021;372:n71. doi: 10.1136/bmj.n71. This work is licensed under CC BY 4.0. To view a copy of this license, visit https://creativecommons.org/licenses/by/4.0/
